# On the Torpidity of Animals

**Published:** 1810-06

**Authors:** Benjamin Smith Barton

**Affiliations:** Philadelphia


### *On the Torpidity of Animals.* By Benjamin Smith Barton, M. D. *of Philadelphia.*

[ Extracted from the Philosophical Magazine. ]

I Lately purchased, and have just finished the reading of, "An Essay on the Torpidity of Animals, by Henry Reeve, M. D." The work has afforded me much amusement, and some instruction; and may, doubtless, be read with great satisfaction and advantage by the younger class of naturalists. It is, however, I think, less replete with new facts and experiments, and with original and enlarged views of the nature and phænomena of TORPID LIFE, than might have been expected, considering the respectable author's opportunities of acquiring information, and the length of time that he has had the subject under his consideration.

Having myself, for several years, been engaged in inquiries relative to the same subject, in various classes of animals, but especially in the Mammalia, the Birds, and the Reptilia (*Amphibia* of Linnæus,) I hope to be able, at no very distant period, to publish the full result of my researches and experiments. I shall then, with that candour, which, I trust, will never forsake me in my inquiries as a naturalist, point out some of the errors (as I now conceive them to be) of Dr. Reeve's work; and, in particular, I shall state, at length, the facts, the actual experiments, and the observations, which compel me to differ from him on some very material questions. At present, I have no other object in view, than to draw your attention, and that of your philosophical readers, to that part of Dr. Reeve's *Essay* which relates to the real or supposed torpidity of birds. This part of his subject, the intelligent author does not seem to have examined with his accustomed ability.

In treating of the "Migration of Birds, Dr. Reeve has the following words: "Here a curious question arises respecting the disappearance of birds. It is singular that this subject should still admit of doubt, when it seems so easy to be decided; yet every month we see queries and answers about the migration of swallows, and every year our curiosity is tempted to be amused with marvellous histories of a party of these birds diving under water in some remote quarter of America. No species of birds, except the swallow, the cuckow, and the woodcock, have been supposed to remain torpid during the winter months. And what is the evidence in favour of so strange and monstrous a suppoa supposition? Nothing but the most vague testimonies, and histories repugnant to reason and experience."*

It appears somewhat surprising to me, that an author who has so long had the subject of the torpidity of animals under his consideration, should have hazarded the assertion contained in the preceding paragraph. Dr. Reeve has, certainly, read of other birds, besides the swallow, the cuckow, and the woodcock, which are said to have been found in a torpid state. And ought he not to have *mentioned* these birds?

In my *Fragments of the Natural History of Pennsylvania*, which Dr. Reeve, if I do not mistake, has seen, for he has referred to the work in his Inaugural Dissertation published in 1803, I have mentioned the common humming-bird *(trochilus colubris)* as one of those American birds which do occasionally become torpid. I have particular reasons for quoting the passage, as it occurs in the *Fragments*. "I have not been able to learn, that the humming-bird winters in any, not even in the warmest parts of the United States. I cannot hesitate to consider it as a bird of passage. A gentleman, however, whose name I do not recollect, wrote a little paper to prove, that these birds continue with us all the winter: why? because one of them was, one frosty day, in the month of October, found a good deal benumbed in a church, in some part of New England, I think in Connecticut."†

In the same work, speaking of the *caprimulgus virginianus*, or Whip-poor-Will of the Americans, I have said: "I have been informed, that some of these birds have been found in a torpid state, in hollow trees, in New-Jersey. But I cannot entirely depend upon the fact; and I have little hesitation in saying, that this bird, as well as the swallows, to which it is allied, is a bird of passage."‡

Here, then, there are two American birds, besides those enumerated by Dr. Reeve, which are supposed, by some persons, to become torpid in the winter season. Nor do these complete the list. It is the opinion of many well-informed persons, in the United States, (but I by no means vouch for the verity of the story,) that the Virginia corn-rake, or rail, *(rallus virginianus,)* becomes torpid, and remains

---

* An Essay, &c. section ii. pages 39, 40.

† Fragments of the Natural History of Pennsylvania, Part First. Appendix I. pages 18 and 19. Philadelphia, 1799.

‡ Fragments, &c. Appendix I. page 18.

remains among the mud and grasses of our meadows, &c. during the winter season. It is asserted, by many other persons, that whole flocks of the Carolina parrot, or parakeet, *(psittacus carolinensis,)* continue in a torpid state, in the hollows of trees, in the state of North Carolina, and in some other parts of the American Union. *I believe entire dependence may be placed upon this statement*; though it would not be difficult to show, that these birds are often seen abroad, and pretty active, when the ground is whitened by snow. I could mention not a few other birds, the torpid state of which has been spoken of by naturalists and others; and these birds I shall mention in my "Facts, Experiments, and Observations, relative to the Torpidity of Animals."

But "what" (says Dr. Reeve) "is the evidence in favour of so strange and monstrous a supposition? Nothing but the most vague testimonies, and histories repugnant to reason and experience."

This, surely, is not the proper language to be employed in the investigation and discussion of physiological questions. Authorities are facts in natural, as well as in civil, history. And in favour of the torpidity of some of the birds which I have mentioned, the authorities are, sometimes at least, highly respectable: nor are they few in number. In regard to the swallows, I shall say but little at present. I have, at this time, in the press, a memoir on the migration and torpidity of these birds. I am confident that I shall be able to convince every candid philosopher, that great numbers of swallows, of different species, do *occasionally* pass into a state of torpidity, more or less profound, not merely "in some remote quarter of America," but in the vicinity of our capital cities, where there are some men of genuine observation and inquiry, and who are as little propense to believe the marvellous in natural history, as any philosophers elsewhere.

I do not suppose, that *all* the swallows of North America become torpid. It is my present opinion, and it was my opinion when I published the "Fragments" in 1799, that the swallows, *in general*, are migratory birds.* But subsequent and very extensive inquiries have convinced me, that the instances of torpid swallows are much more frequent than I formerly supposed they were; and that there

---

* See Fragments, &c. Appendix I. page 16. See, also, Introduction to this work, pages xii and xiii. § xxiv, xxv, xxvi.

there are two species of the genus *Hirundo*, which are peculiarly disposed to pass the brumal season in the cavities of rocks, in the hollows of trees, and in other similar situations, where they have often been found in a *soporose* state. These species are the *hirundo riparia*, or sand-swallow, commonly called, in the United States, bank-swallow and bank-martin; and the *hirundo pelasgia*, or aculeated swallow, which we call chimney-bird and chimney-swallow. *There is no fact in ornithology better established, than the fact of the occasional torpidity of these two species of Hirundo.*

I say nothing of the torpidity of swallows, " under water." But I do not wholly deny *this* fact. And I take much pleasure in referring Dr. Reeve to a short paper, in the *Transactions of the American Philosophical Society*, vol. vi. part i. relative to the discovery of a torpid swallow under a quantity of mud and leaves. The author of that paper was a most worthy and respectable man; and a man so religiously attached to truth, that I believe him to have been incapable of uttering a falsehood. He was, moreover, a man of nice observation, and of a philosophical turn of mind.

I do not wish to urge this part of the swallow's history any further. I have nothing to say in support of the " swallow song." But when, in page 44, Dr. Reeve asserts, that no swallows " were ever found in all the rivers and lakes of England, Wales, Ireland, Scotland, or Switzerland, although fishermen are constantly employed on these their supposed hiding places," does he mean to say, that it has never been asserted by any of his countrymen, that swallows have been found torpid, under water, *in England?* Swallows are said to have been found torpid " in the river Thames;" and the fact seems to have been credited by some illustrious Englishmen in the 17th century; and among others, if I do not mistake, by the immortal William Harvey.*

But

* In Dr. Birch's *History of the Royal Society*, vol. iv. there are some curious notices about swallows. The following may not be deemed wholly unworthy of Dr. Reeve's attention. " Sir John Hoskyns proposed, that it might be duly examined, what becomes of the swallows, and in what state they are during the winter. In answer to which Mr. Henshaw affirmed, that the chancellor of Denmark told him, as an undoubted truth, that in Iceland, there had been taken out of the ice swallows, which being afterwards brought into a warm stove, recovered and flew about the room."—Mr. Henshaw observed, " that he had an account like the former concern-

 ing

But I will take my leave of the swallows. Since I published my *Fragments*, I have obtained much information relative to the torpidity of the humming-bird. I have hinted at this subject, and have, indeed, most pointedly admitted the fact, in my letter to Mons. la Cepede, published in your Philosophical Magazine. I am now fully persuaded, that instances of the torpidity of the *trochilus* are by no means uncommon in the United States: and I regret my having treated with so little respect, the opinion of the Connecticut gentleman already alluded to. It is certain, at least, that the *trochilus*, like the generality of the swallows, is very impatient of cold; and that it sometimes even in our houses, very suddenly passes into a profound slumber, from which, however, it awakes, to enjoy all the privileges of its life. I say this is *certain*. And this, so far as his sentiments may be collected from his *Essay*, is more than Dr. Reeve is willing to admit of *any* species in the great class of birds.

The fact of the torpidity of the *trochilus* was not unknown above two centuries ago. It is related by the Spanish historians Herrera, Ximenes, and several others, tho' it must be confessed that these writers have mixed with the truth, some *fable*. I have lately conversed with an intelligent gentleman, who was born, and has long resided, in the kingdom of Mexico. He assures me, that the fact of the torpidity of the *trochilus* is known to every one in that country, and the adjacent provinces. He added, that he had himself seen one of these little birds in its brumal sleep, in a tree. I shall discuss this subject at length, and shall illustrate it by actual experiments, in my work on the torpid state of animals, to which I have already alluded. In the mean while, I flatter myself that the following lines, a part of which immediately relates to the *somnus* of the *trochilus*, will not be wholly unacceptable to some ing swallows from our watermen, viz. that they have found them in the river Thames; and that towards the end of the year they assemble in great numbers on the little islands of the river, and then submerge themselves in the water."—"Upon reading the minutes of the last meeting, Mr. Henshaw remarked, that Dr. Harvey had considered the state of swallows in the winter, and had dissected some of them, which had been found under water, and could not observe that there was either warmth or motion in them." —"Mr. Chetwynd, of Ingstree, being present (at a meeting of the Royal Society), observed, that during the time that the swallows are laid up for the winter, they moult, and return in the spring with all new feathers." The History of the Royal Society of London, &c. &c. By Thomas Birch, D.D. Secretary to the Royal Society, vol. iv. pages 533, 534, 537.

some of your readers. The author is Raphael Landivar, a native of Guatimala; and his poem, entitled *Rusticatio Mexicana,* in fifteen books, besides an Appendix, in verse also, deserves to be much better known than it appears to be. It is, indeed, well worthy of an English translation; and I sincerely wish that the elegant Mr. Sotheby, whose translation of the *Georgics* of Virgil has so deservedly procured him a high reputation, could be induced to undertake the task. My copy of Landaver's work, which is, I believe, a very rare one, would be at his service. The public pulse might be tried by the publication of a version of one or two of the books.

In his 13th book the author treats of birds. And here it is that he speaks of the humming-bird, its manners, its sleep, &c.

"Nil tamen exiguo novit præstantius orbis
*Colibrio* dulcis spoliato murmure vocis,*
Sed claro tenues pennâ radiante per artus.
Exiguum corpus, forsan non police majus,
(Quod rostro natura parens munivit acuto
Atque artus ferme totos æquante volucris.)
Induit aurato viridantes lumine plumas,
Et varios miscet tractos a sole colores.
Ille volat rapidum Zephyrum superante volatu,
Et raucum pennâ tollit stridente susurrum.
Roscida si vero fragranti educere flore
Mella velit rostro, viresque reducere membris,
(Quippe aliâ quacumque negat se pascere mensâ)
Sistitur in medio concussis aëre pennis,
Nectareum donec tereti trahat ore liquorem.
Ast adeo prompte subtiles concutit alas,
Ut vigiles fugiant oculos, ludantque citatæ;
Suspensamque putes volucrem super æthera filo.
Sin autem sylvis borealis bruma propinquet,
Plusque vagus solito frigescat Jupiter imbre,
Frigida præcipiti linquit *Colibrius* arva
Nostra fugâ, linquitque levi viridaria pennâ,
Et longum montis nigris absconditus umbris
Indulget placido, ceu Progne arguta, sopori,
Dum luces Aries stellatis noctibus æquet,
Verque novum pratis antiquum reddat hororem."

*Rusticatio Mexicana,* lib. xiii. v. 217—242.

 All

* Avicula hæc *Colibri,* in America Meridionali, in Septentrionali vero *Chupa-mirto* dicitur." Note by Landivar.

All this, Dr. Reeve will perhaps say, may do very well in poetry: but something more positive on the subject of the "placidus sopor" of the colibri is required. Some facts, and therefore something more positive, I have already mentioned: and many additional facts, with experiments, I promise to give in another place. At present I will only add, that Mr. Landivar mentions the torpidity of the humming-bird, not as a fable, but as an established truth. For in the short *Monitum* prefixed to his interesting work he says, "In hoc autem opusculo nullus erit fictioni locus, eam si excipias, quæ ad lacum Mexicanum canentes poetas inducit. Quæ vidi refero, quæque mihi testes oculati, cæteroquin veracissimi, retulere. Præterea curæ mihi fuit oculatorum testium auctoritate subscripta, quæ rariora sunt, confirmare."

*Philadelphia, Nov.* 1, 1809.

